# Protection Against Arthritis by the Parasitic Worm Product ES-62, and Its Drug-Like Small Molecule Analogues, Is Associated With Inhibition of Osteoclastogenesis

**DOI:** 10.3389/fimmu.2018.01016

**Published:** 2018-05-14

**Authors:** James Doonan, Felicity E. Lumb, Miguel A. Pineda, Anuradha Tarafdar, Jenny Crowe, Aneesah M. Khan, Colin J. Suckling, Margaret M. Harnett, William Harnett

**Affiliations:** ^1^Strathclyde Institute of Pharmacy and Biomedical Sciences, University of Strathclyde, Glasgow, United Kingdom; ^2^Institute of Infection, Immunity and Inflammation, University of Glasgow, Glasgow, United Kingdom; ^3^Department of Pure and Applied Chemistry, University of Strathclyde, Glasgow, United Kingdom

**Keywords:** ES-62, immunomodulation, osteoclast, parasitic worm, rheumatoid arthritis

## Abstract

The immunomodulatory actions of parasitic helminth excretory-secretory (ES) products that serendipitously protect against development of chronic inflammatory disorders are well established: however, knowledge of the interaction between ES products and the host musculoskeletal system in such diseases is limited. In this study, we have focused on ES-62, a glycoprotein secreted by the rodent filarial nematode *Acanthocheilonema viteae* that is immunomodulatory by virtue of covalently attached phosphorylcholine (PC) moieties, and also two synthetic drug-like PC-based small molecule analogues (SMAs) that mimic ES-62’s immunomodulatory activity. We have previously shown that each of these molecules prevents development of pathology in collagen-induced arthritis (CIA), a model of the musculoskeletal disease rheumatoid arthritis (RA) and reflecting this, we now report that ES-62 and its SMAs, modify bone remodeling by altering bone marrow progenitors and thus impacting on osteoclastogenesis. Consistent with this, we find that these molecules inhibit functional osteoclast differentiation *in vitro*. Furthermore, this appears to be achieved by induction of anti-oxidant response gene expression, thereby resulting in reduction of the reactive oxygen species production that is necessary for the increased osteoclastogenesis witnessed in musculoskeletal diseases like RA.

## Introduction

The musculoskeletal disease rheumatoid arthritis (RA) is a chronic, autoimmune inflammatory condition that results in the debilitating loss of articular bone in affected joints (1). Inflammation in such joints is orchestrated by the hyper-proliferative responses of synovial fibroblasts and infiltration of activated leukocytes, resulting in bone erosion that can be present prior to manifestation of clinical disease (2). Cells associated with bone remodeling such as osteoclasts (OCs), and also immune system cells including T and B lymphocytes, rely on cytokines like Receptor Activator of NF-κB Ligand (RANKL), osteoprotegerin (OPG), and pro-inflammatory mediators to control cell maturation and pathogenic responses. These events occur in the lymph node and bone marrow (BM) and additionally drive OC differentiation at bone-remodeling sites in arthritic joints (3). Thus, in patients with RA, synovial fibroblasts and T cells produce increased levels of RANKL and interleukin (IL)-17 compared to healthy controls, leading to the dysregulated differentiation of OCs at the articular surface, and consequently resulting in erosion and deformation of bone (4, 5).

This aberrant osteoclastogenesis contrasts with the tightly regulated interactions between OCs and their bone-forming partners, osteoblasts (OBs), that continuously act to maintain homeostatic renewal of bone (6). In this case, OCs differentiate from OC precursors (OCPs) in the monocyte lineage and are recruited to the bone, where OBs produce macrophage-colony stimulating factor (M-CSF) and RANKL to stimulate terminal OC differentiation (3). The interaction between RANKL and its receptor RANK activates the transcription factor NFATc1 which controls the production of DC-STAMP and cathepsin K that are essential for the fusion and function of mature bone-eroding cells (7–10). In addition, the process of osteoclastogenesis is reliant on the production of reactive oxygen species (ROS) as both M-CSF and RANKL signaling induce ROS to enhance OC differentiation and moreover, treatment with *N*-acetyl-l-cysteine (NAC), a scavenger of ROS, inhibits the differentiation of OCs preventing RANKL-induced ROS (11, 12). Importantly, scavenging ROS with the aid of NAC in synovial fibroblasts isolated from RA patients results in their decreased RANKL production and inhibited Th17 differentiation (13).

Although the immunomodulatory properties of parasitic helminths and their excretory–secretory (ES) products in modulating inflammatory diseases including RA are well documented, the effects of the helminths or their ES products on bone during such conditions remains poorly studied. Among the most widely characterized helminth ES products is the phosphorylcholine (PC)-containing glycoprotein ES-62, produced by *Acanthocheilonema viteae* (14, 15). It is well-established that ES-62, *via* its PC moieties, prevents the development and progression of arthritic disease, with histopathological analysis demonstrating that it not only suppresses inflammatory cell infiltration of the joints, but also reduces cartilage and bone damage, in the murine model of RA, collagen-induced arthritis (CIA) (14, 16, 17). Specifically, ES-62 promotes IL-22-mediated tissue repair responses in the joint, while disrupting Th17 and IL-17-producing γδ T cell inflammatory networks to facilitate the resolution of disease (16, 17). Likewise, drug-like small molecule analogues (SMAs) based on the active PC moiety of ES-62 mimic both the anti-inflammatory and bone protective effects of ES-62 and thereby prevent the development and progression of joint damage associated with arthritis in CIA (18–20).

We, therefore, aimed to investigate whether these helminth-derived agents could also impact on pathogenic osteoclastogenesis to resolve inflammation and joint damage in the CIA model. In doing so, we have established that treatment with ES-62 can alter BM monocyte populations *in vivo* and reduce functional OC differentiation *in vitro*. We also demonstrate that the drug-like SMAs based on this helminth product, mimic ES-62’s ability to modulate OC differentiation *in vitro* by orchestrating an anti-oxidant response that suppresses ROS production.

## Materials and Methods

### Animals

Male 6- to 8-week-old C57BL/6 mice were bred and maintained under specified pathogen-free and standard *ad libitum* conditions at the University of Strathclyde’s Biological Procedures Unit. Male 6- to 8-week-old DBA/1 mice (Envigo; Bicester, UK) were housed and maintained in the Central Research Facility of the University of Glasgow. All experiments were approved by, and conducted in accordance with, the Animal Welfare and Ethical Review Boards of the Universities of Strathclyde and Glasgow and UK Home Office Regulations and Licenses PIL I518666F7, PPL 60/4314, PPL P8C60C865, PIL 1675F0C46, and PIL ICEBDB864.

### Collagen-Induced Arthritis

Collagen-induced arthritis (CIA) was induced in male DBA/1 mice (8- to 10-week-old; ~ 20 g) following intradermal immunization with 100 µg of bovine collagen type II (CII) emulsified with complete Freud’s adjuvant (MD Biosciences) on day 0 with a further injection of 200 µg CII in PBS given intraperitoneally on day 21 as described previously (14). Animals were treated *via* the subcutaneous route with either PBS or 2 μg/injection of ES-62 (~ 0.1 mg/kg) on days −2, 0, and 21 and joint inflammation was scored as previously described (14). Disease articular scores were assessed daily as 0 (normal), 1 (erythema), 2 (erythema plus swelling), 3 (extension of swelling), or 4 (loss of function), with the overall score being the sum of those from all four limbs: animals were culled when indicated or at least one of the PBS control group had reached a score of 10 or clinical symptoms developed in all four limbs. Joint damage was also routinely assessed by histopathology. Typically, disease incidences ≥80%, where incidence is defined as the percentage of animals achieving an articular score ≥1, are achieved by about day 30, with articular scores generally plateauing around this time (14, 16–21). Purified endotoxin-free ES-62 and SMAs 11a, 12b, and 19o were prepared as previously described (14, 18).

### Histology

Paws from CIA mice were fixed in 4% paraformaldehyde prior to decalcification and paraffin wax embedding for H&E staining (7 µm sections) following a standard protocol. For cathepsin K detection by immunofluorescence, sections were cleared in histoclear and rehydrated before antigen retrieval in citrate buffer for 20 min at 95°C. Sections were blocked in 10% FBS in PBS for 30 min at room temperature (RT), endogenous avidin/biotin was quenched (Vector Laboratories, UK), and sections incubated overnight at 4°C with rabbit anti-mouse cathepsin K (Abcam, UK). Sections were then incubated for 60 min at RT with biotinylated goat anti-rabbit IgG (Vector Laboratories, UK) and followed with streptavidin-conjugated Alexa Fluor 647 (Vector Laboratories, UK) for 30 min at RT. DAPI (1 µg/ml) was used as a nuclear stain (Sigma-Aldrich, UK). Sections were washed in PBS with 0.05% (v/v) Tween-20 between each incubation. Following staining, sections were dehydrated through ethanol, mounted, and cathepsin K^+^ multinucleated cells on the bone surface were enumerated using an EVOS FL Auto Cell Imaging System.

### Flow Cytometry

BM cells were suspended in FACS buffer (2.5% BSA; 0.5 mM EDTA, in PBS) following red cell-lysis with 0.8% NH_4_Cl buffer. Phenotypic surface markers were labeled using PE-conjugated anti-CD3/B220/Ter119, FITC anti-CD11b, PerCP-Cy5.5 anti-Ly6C, APC anti-Ly6G or CD11b, and Biotin anti-CD115 antibodies and APC Cy7 conjugated streptavidin (Biolegend). Cells were stained with 7AAD (BD Bioscience, UK) to assess cell death. For hematopoietic stem cell (HSC) analysis, PE-conjugated lineage cocktail and PE-conjugated isotype antibodies were used alongside FITC anti-Sca-1 and APC anti-CD117 antibodies. Data were acquired using a FACS Canto flow cytometer and analyzed using FlowJo Software (Tree Star Inc, OR, USA, version 8.8.7) and populations were gated using isotype and fluorescence minus one controls.

### *In Vitro* OC Differentiation

Osteoclasts (OCs) were differentiated from naïve C57BL/6 or DBA/1 mice that were either naïve control animals or DBA/1 mice that had undergone CIA and received either PBS or ES-62 treatments, *in vivo*. BM was flushed from the tibias and femurs of these mice using a sterile 23G needle and syringe in PBS and aspirated to create a single cell suspension. This single cell suspension was then passed through a 20 µm cell strainer prior to centrifugation at 400 × *g* and re-suspension in PBS. Following cell counting, single cells were washed and re-suspended at 1 × 10^6^/ml in “complete” αMEM medium (containing 50 U/ml penicillin, 50 µg/ml streptomycin, and 10% FCS) and 9 ml of cell suspension were incubated overnight with 30 ng/ml M-CSF (Peprotech, London, UK). Following overnight incubation at 37°C in 5% CO_2_, non-adherent BM cells were removed, counted, and re-suspended in fresh complete αMEM medium supplemented with 30 ng/ml M-CSF and 50 ng/ml RANKL at 0.5 × 10^6^ cells/ml. These non-adherent BM cells (200 µl) were then seeded in 96-well tissue-culture plates to initiate OC differentiation. Preliminary experiments established that while treatment of cells with phosphorylcholine conjugated to bovine serum albumin (PC-BSA) from day 1–4 did not inhibit the numbers of OCs generated, exposure to PC-BSA at day 4 resulted in inhibition of the OC fusion underpinning generation of large multinucleated OCs (data not shown). Thus medium was refreshed on day 4 and treated with vehicle, ES-62, bovine serum albumin (BSA—Sigma-Aldrich, UK), phosphorylcholine conjugated to BSA (PC-BSA) (22), phosphorylcholine chloride (PC—Sigma, UK), or SMAs 11a, 12b, or 19o, at the indicated concentrations, for 24 h prior to assessment of functional maturation. Cells were assessed for OC differentiation by TRAP staining (Leukocyte Acid Phosphatase Kit, Sigma, UK) on days 4, 5, and 6 as indicated and cells that stained positive for TRAP with ≥3 nuclei were counted as OCs. The average size of individual OCs per field of view (FOV) was calculated using Image J software. Images were obtained on an EVOS FL Auto Cell Imaging System or a Leica DM IL LED at 4× magnification with scale bars set at 1,000 µm.

### *In Vitro* Bone Resorption Assay

Dentine Discs (Immunodiagnostics Systems Ltd., UK) were used to measure the functional capacity of differentiated OCs. Briefly, non-adherent BM was cultured on dentine discs for 14 days with medium refreshed every fourth day. Vehicle controls and treatments were commenced on day 4 and added with every medium replacement. OCs were lysed on dentine discs by incubation with distilled H_2_O prior to visualization of resorption pits using black ink. The area of resorption was measured as a percentage of the total area using ImageJ software. Images were obtained on an EVOS FL Auto Cell Imaging System or a Leica DM IL LED at 10× magnification with scale bars set at 400 µm.

### qRT-PCR

BM cells (10^6^), or OCs that were pre-treated with SMAs (5 µg/ml) for 24 h, were lysed in RNeasy lysis buffer prior to mRNA extraction using an RNeasy Plus Mini kit (Qiagen, Germany) according to manufacturer’s instructions. Additionally, cleaned and flushed femurs and tibias were then flushed with QIAzol to obtain RNA from bone-lining cells as previously described (23). cDNA was generated using a high capacity cDNA Reverse Transcriptase kit (Applied Biosystems, Life Technology) before use in PCR amplifications using the StepOne Plus™ real-time PCR system (Applied Biosystems). KiCqStart^®^ qPCR Ready Mix (Sigma-Aldrich) was used in conjunction with the following primer pairs: osteopontin (OPN) (*spp1*; forward—GGATGAATCTGACGAATCTC, reverse—GCATCAGGATACTGTTCATC), RANK (*tnfrsf11a*; Forward—GAAATAAGGAGTCCTCAGGG, reverse—TAGAATCTCTGACTTCTGCC), RANKL (*tnfsf11*; forward—TCTGTTCCTGTACTTTCGAG, reverse—TTCATGGAGTCTCAGGATTC), OPG (*tnfrsf11b*; forward—GAAGATCATCCAAGACATTGAC, reverse—TCCTCCATAAACTGAGTAGC), DC-STAMP (*dcstamp*; forward—TCTGCTGTATCGGCTCATCTC, reverse—ACTCCTTGGGTTCCTTGCTT), Cathepsin K (*ctsk*; forward—CAGAGGGTACAGAGAGATTC, reverse—TCATCATAGTACACACCTCTG), MMP9 (*mmp9*; forward—CTTCCAGTACCAAGACAAAG, reverse—ACCTTGTTCACCTCATTTTG), NFATc1 (*nfatc1*; forward—CCAAGTCAGTTTCTATGTCTG, reverse—ATAATTGGAACATTGGCAGG), β3 (*itgb3*; forward—TATAGTGAGCTCATTCCTGG, reverse—ATTTTCCCGTAAGCATCAAC), and β-actin (*actb*; forward—GATGTATGAAGGCTTTGGTC, reverse—TGTGCACTTTTATTGGTCTC). TaqMan™ master mix (Applied Biosystems) was used to analyze the following set of genes; heme oxygenase-1 (HMOX1) (*Hmox1*—Mm00516005_m1), GCLM (*gclm*—Mm00514994_m1), GCLC (*gclc*—Mm00802655_m1), and GAPDH (*gapdh*—Mm99999915_g1). Data were normalized to the reference genes GAPDH or β-actin to obtain the ΔCT values that were used to calculate the fold-change from the ΔΔCT following normalization to the biological control group.

### ROS Assay

The presence of ROS was measured on day 5 of the OC differentiation assay, following 24 h pre-treatment with SMAs, using 2′,7′-dichlorofluorescein diacetate (DCF-DA, Sigma, UK). Briefly, to measure ROS, cells were washed in pre-warmed PBS twice prior to incubation with 100 µl of 50 µM DCF-DA for 30 min at 37°C. Following washing in PBS, fluorescence was detected at 492 nm excitation and 529 nm emission wavelengths. Unstained cells were used to control for background fluorescence and biological replicates were normalized to controls.

### Statistics

All data were analyzed using GraphPad Prism 6 software. For parametric data, Student’s *t*-test or one-way ANOVA with Bonferroni’s or Fishers LSD post-tests were employed. Non-parametric data were analyzed using Kruskal–Wallis test and Dunn’s post-test or Mann–Whitney *U* test. Indicators of significance include *p < 0.05, **p < 0.01, and ***p < 0.001.

## Results

### ES-62 Normalizes the BM OC Progenitor Populations in Arthritic Animals

**Figure 1:**
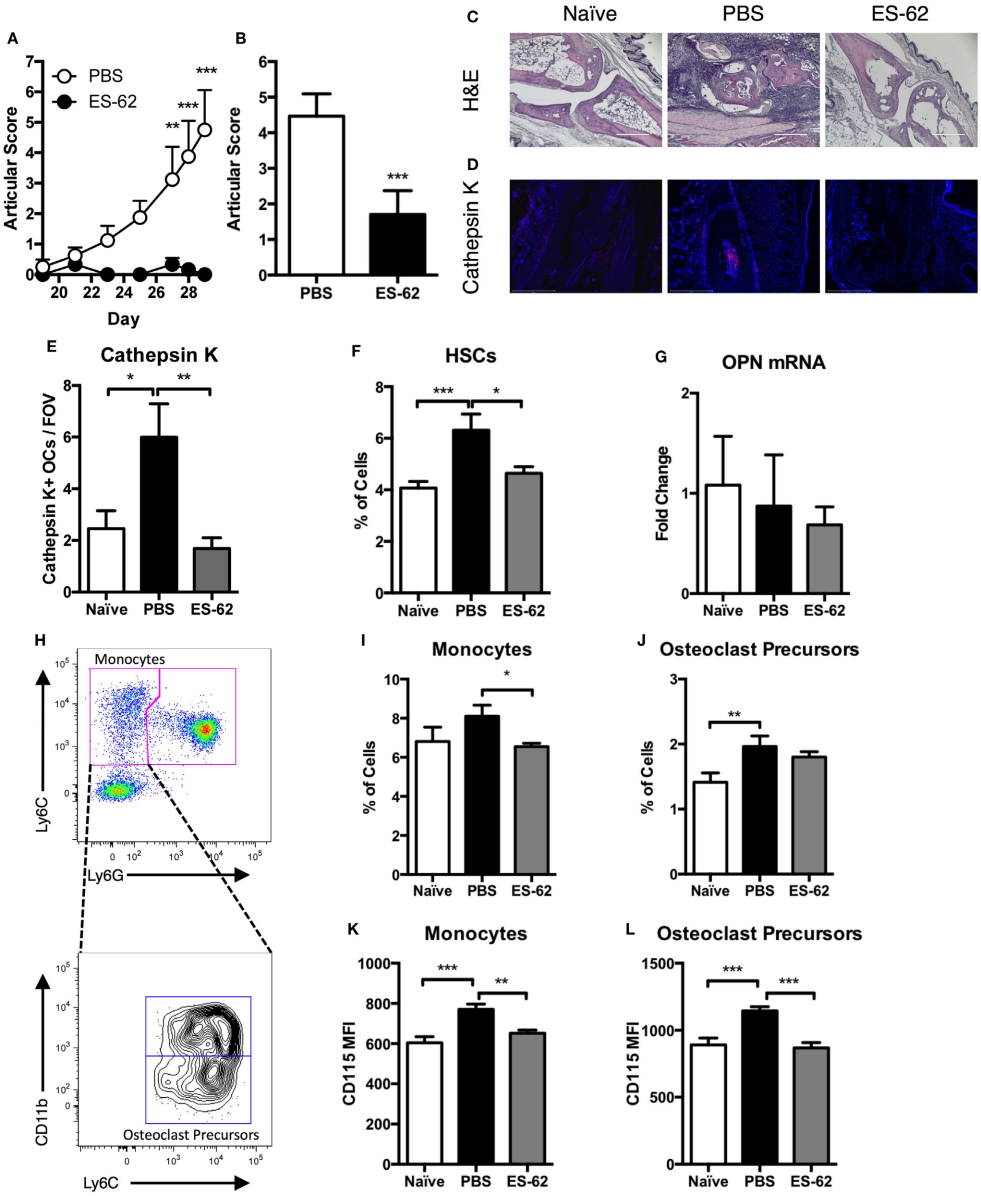
ES-62 alters the progenitor populations in the BM of arthritic animals. Arthritic scores of DBA/1 mice undergoing CIA from challenge until cull at day 29 (PBS-treated CIA animals, *n* = 8; ES-62-treated CIA animals, *n* = 6) **(A)** and the scores of all animals used in the study obtained from four independent CIA models at cull [PBS-treated (*n* = 30) and ES-62-treated (*n* = 20) CIA animals] **(B)**. Joint sections of representative animals (PBS-CIA, score 7 and ES-62-CIA, score 0) in each treatment group were subjected to histological analysis employing H&E staining [**(C)** scale bar = 400 µm] and analysis of expression of cathepsin K by immunofluorescence [**(D)** DAPI, blue; cathepsin K, red; scale bar = 500 µm]. The number of cathepsin K^+^ multinucleated cells within the joints of animals [naïve (*n* = 4), PBS (*n* = 3), and ES-62 (*n* = 4); three field of view (FOV) per sample] were enumerated **(E)**. BM was taken from naïve (*n* = 8), PBS (*n* = 12), and ES-62 (*n* = 7) treated CIA animals to analyze the frequency of hematopoietic stem cells (HSCs—Lineage^neg^Sca-1^+^CD117^+^) **(F)**. QIAzol lysates of bone-lining cells from the femur and tibia of animals were analyzed for osteopontin (OPN) mRNA (naïve, *n* = 4; PBS, *n* = 8; ES-62, *n* = 7) **(G)**. BM from CIA animals was analyzed by flow cytometry for the phenotypic markers of monocytes and OC precursors (OCPs) following gating of single CD3^−^B220^−^Ter119^−^ cells. The gating strategy for distinguishing monocytes from neutrophils is demonstrated (Ly6C^high^Ly6G^−^ population) and this is subsequently used to identify the OCPs (CD11b^low^Ly6C^high^Ly6G^−^ population) that reside within the global monocyte population **(H)**. The percentage of monocytes and OC precursors **(I,J)** from naïve (*n* = 24), PBS-treated [*n* = 26 for **(I)** and 30 for **(J)**], and ES-62-treated (*n* = 19) CIA animals as well as the expression of CD115 on the surface of monocytes and OC precursors [(**K,L**); naïve, *n* = 4; PBS, *n* = 8; ES-62, *n* = 7] was examined. Data are pooled from four independent experiments and are presented as mean values of individual mice ± SEM. Two-tailed Mann–Whitney *t*-test or Kruskal–Wallis with Dunn’s multiple comparisons was used for non-parametric data and one-way ANOVA with Fishers LSD was used to analyze parametric data where *p < 0.05, **p < 0.01, and ***p < 0.001.

In line with our previously published observations (14), DBA/1 mice treated with ES-62 were protected from developing arthritis in the CIA model both in terms of articular score (Figures 1A,B) and disease incidence (Figure 1A: PBS, 87.5%; ES-62, 0%, at cull). This impact of ES-62 on disease pathology was confirmed by H&E staining of joint sections (Figure 1C), which showed that exposure to the parasite product reduced inflammatory cell infiltration and cartilage and bone destruction. Additionally, upon analysis of representative animals from each group, the levels of cathepsin K^+^ OCs found in such sections were decreased in the paws of ES-62-treated animals compared to those of PBS-treated CIA animals, returning to the levels found in joints from naïve healthy DBA/1 mice (Figures 1D,E). To understand the effect of inflammatory arthritis on BM progenitors we identified hematopoietic stem cells (HSCs) within the BM of animals undergoing CIA. Analysis of Sca-1^+^CD117^+^ cells within the Lineage^neg^ population showed that PBS-treated CIA animals had an increased proportion of HSCs in the BM compared to both naïve and ES-62-treated CIA animals (Figure 1F). The BM HSC population is supported and maintained by stromal and OB production of OPN, but analysis of OPN mRNA in the bone-lining cells (23) of the femurs and tibias showed that OPN mRNA levels were not significantly different in PBS-treated CIA mice relative to naïve and ES-62-treated CIA animals (Figure 1G). As ES-62 is able to return HSC levels to those found in healthy animals and show evidence of reducing cathepsin K^+^ OC numbers in the joints of arthritic mice, we next aimed to determine specifically whether CIA would affect defined OC progenitor populations in the BM. Due to their differentiation from the myeloid lineage, we focused on phenotypic analysis of both monocyte (CD3^−^B220^−^Ter119^−^Ly6G^−^Ly6C^high^) and OCP (CD3^−^B220^−^Ter119^−^Ly6G^−^Ly6C^high^CD11b^low^) populations (Figure 1H), as OCPs constitute a small population of cells within the umbrella of monocyte heterogeneity, as defined by Charles et al. (24). Our analysis showed that treatment with ES-62 reduced the absolute percentage (%) of monocytes within the BM of mice undergoing CIA (Figure 1I). Moreover, CIA (PBS) increased the % of OCPs in the BM compared to naïve animals (Figure 1J): although treatment with ES-62 was unable to significantly alter the level of BM OCP populations observed from that in PBS-treated CIA animals, the % of OCPs present in ES-62-treated CIA animals was not significantly different from that of naïve animals, suggesting that ES-62 was having some effect in reducing the % of these progenitor cells in BM of CIA animals. In addition, ES-62 treatment was able to decrease the expression of CD115 (the M-CSF receptor) on the surface of both monocytes (Figure 1K) and OCPs (Figure 1L) compared to PBS-treated CIA animals, an effect that would decrease their ability to differentiate into OCs during inflammation.

### ES-62 Alters the Functional Differentiation of OCs and the Bone-Remodeling Axis in Arthritic Animals

**Figure 2:**
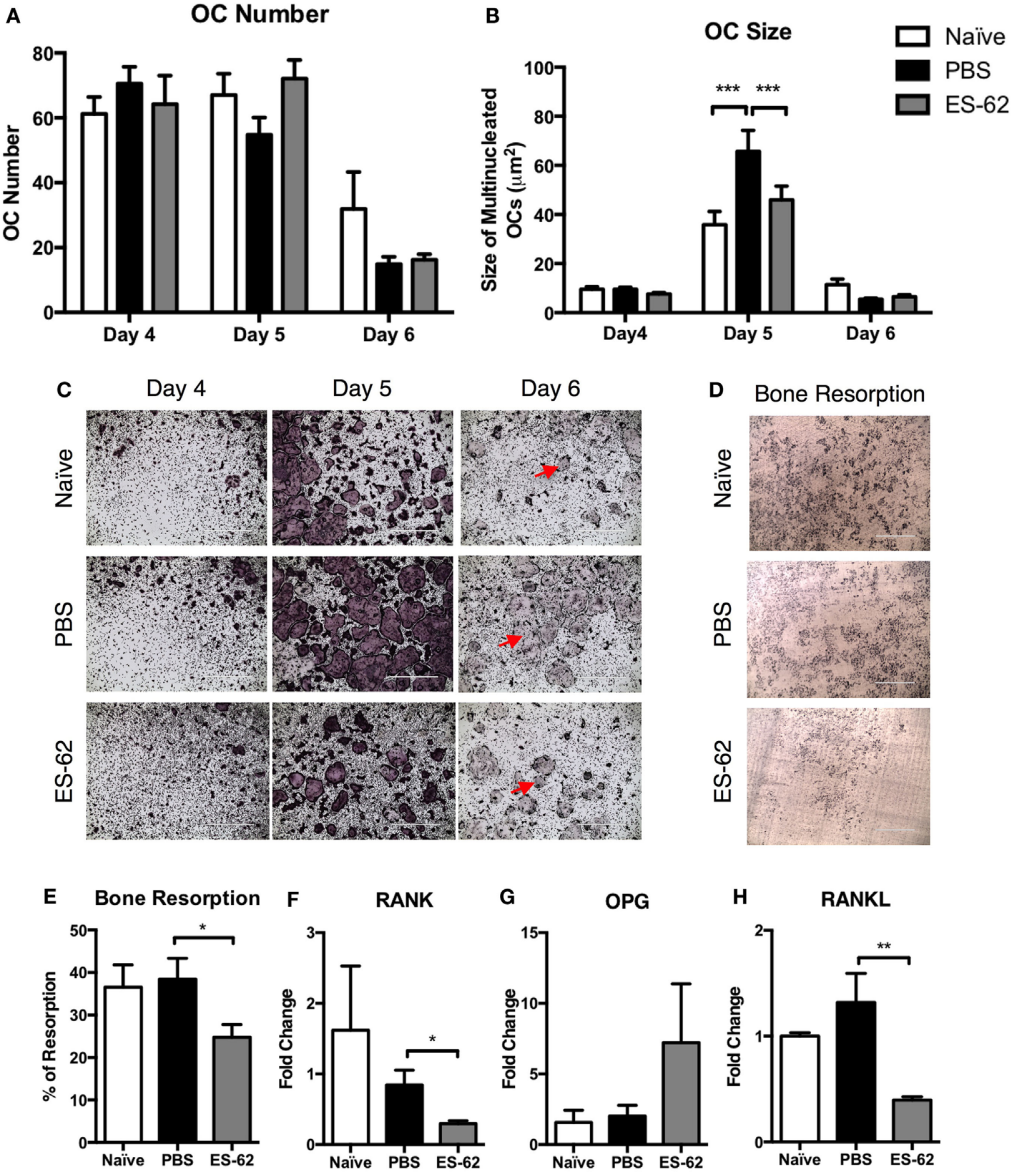
Exposure to ES-62 *in vivo* alters the *ex vivo* responses of OC progenitors and decreases RANKL mRNA in the bone marrow (BM) of arthritic animals. Non-adherent BM, naïve (*n* = 3), PBS-treated CIA (*n* = 3), and ES-62-treated CIA (*n* = 3) from DBA/1 mice, was harvested at cull and cultured for 4–6 days with 30 ng/ml M-CSF and 50 ng/ml RANKL. Medium was refreshed on day 4 and OC differentiation was assessed by TRAP staining. The number **(A)** and size **(B)** of live TRAP^+^ multinucleated OCs with >3 nuclei were calculated using ImageJ analysis software. Representative images of OCs (PBS-CIA, score 10 and ES-62-CIA, score 0) at each time point are provided with red arrows indicating dead OCs [**(C)** scale bar = 1,000 µm]. Data are from one experiment performed in triplicate presented as mean ± SD and are representative of two experiments, although the kinetic profile of OC differentiation was observed with BM from three mouse models comparing naive and PBS-CIA mice. Representative images of dentine slices following 14-day culture with OCs from naïve (*n* = 3), PBS (mean score 6; *n* = 4), or ES-62 (mean score 3; *n* = 4)-treated CIA-animal BM [**(D)** scale bar = 400 µm]. ImageJ software was used to calculate the % of resorption and data are presented as mean ± SEM of individual mice performed in triplicate **(E)**. mRNA transcripts from naïve (*n* = 4), PBS-treated CIA (*n* = 8), and ES-62-treated CIA (*n* = 7) animals, collected at cull, were analyzed for **(F)** RANK (*tnfrsf11a*), **(G)** OPG (*tnfrsf11b*), and **(H)** RANKL (*tnfsf11*). Data from a single experiment are shown as mean values (of triplicate analyses) ± SEM of individual mice and are representative of four experiments. Two-way ANOVA with Fisher’s LSD post-test was used to analyze experimental conditions comparing PBS-treated CIA with naïve and ES-62-treated CIA animals, where ***p < 0.001. Kruskal–Wallis with Dunn’s post-test was used to analyze non-parametric data, where *p < 0.05 and **p < 0.01.

Previously, exposure to ES-62 was found to alter differentiation of macrophages from BM progenitors (25) and, therefore, given its effects on OCPs, we next aimed to investigate whether OC differentiation from ES-62-treated CIA-mouse BM was altered compared to that from PBS-treated CIA animals. OCs are short-lived cells with their apoptosis playing roles in regulating osteoclastogenesis (26, 27): thus, due to the dynamic nature of their differentiation and cell death (26), a time course of OC differentiation was performed to observe the effects of *in vivo* exposure to ES-62 on the kinetics and extent of differentiation of OCs derived from BM of mice undergoing CIA. As shown in Figures 2A,B, by day 4 comparable levels of osteoclastogenesis in terms of OC number and size were observed among treatment groups: however, by day 5, there was a significant increase in cell fusion associated with CIA resulting in a decrease in cell number, but an increase in cell size in the PBS-treated CIA animals compared to naïve and ES-62 treated animals. By the sixth day of culture the majority of large multinucleated cells have begun to apoptose (Figure 2C) with similar levels of live cells remaining in the PBS- and ES-62-treated groups. Representative images of OC differentiation are provided with cell death indicated by red arrows (Figure 2C). To assess the functional capacity of the BM progenitors from naïve and PBS- and ES-62-treated CIA animals, the BM was cultured for 14 days on dentine slices, revealing that ES-62-CIA BM had a reduced ability to resorb bone compared to PBS-CIA BM (Figures 2D,E).

In order to further understand how the inflammatory milieu in arthritic animals influences the OC progenitor populations and the differentiation potential of OCs, the RANK/OPG/RANKL bone-remodeling axis was analyzed. Whole BM lysates, collected at cull, were used to analyze the mRNA transcripts of RANK (*tnfrsf11a*), OPG (*tnfrsf11b*), and RANKL (*tnfsf11*) to identify potential changes that occur during disease and that may be (differentially) modified following treatment with ES-62. As shown in Figure 2F, the mRNA level for RANK was not significantly changed in PBS-treated CIA animals compared to naïve controls. However, treatment with ES-62 resulted in a decrease in RANK transcript. Similarly, OPG mRNA levels were unchanged in PBS-treated animals compared to naïve animals, however, ES-62 treatment increased OPG mRNA in the BM of animals, although this did not reach statistical significance (Figure 2G). No changes were observed in RANKL mRNA levels between naïve and PBS-treated animals, while ES-62 treatment strikingly decreased RANKL mRNA compared to PBS-treated CIA animals (Figure 2H).

Overall, the data in Figures 1 and 2 indicate that arthritic disease in DBA/1 animals undergoing CIA alters both the levels of OC progenitor populations and their potential to form large multinucleated OCs, while the helminth product ES-62 acts to restore the level of OCPs to that resembling BM OCPs from naïve DBA/1 animals and inhibit their functional maturation. While the RANK/RANKL/OPG bone-remodeling axis is not substantially altered during CIA, the changes in RANK/RANKL/OPG mRNA induced by ES-62 treatment may account for the modulation of progenitor populations and their subsequent capacity to undergo osteoclastogenesis and indicate that ES-62 does not simply prevent the rewiring of progenitors occurring in the naïve to CIA transition.

### ES-62 Modulates Differentiation of Osteoclast Functional Responses *In Vitro*

**Figure 3:**
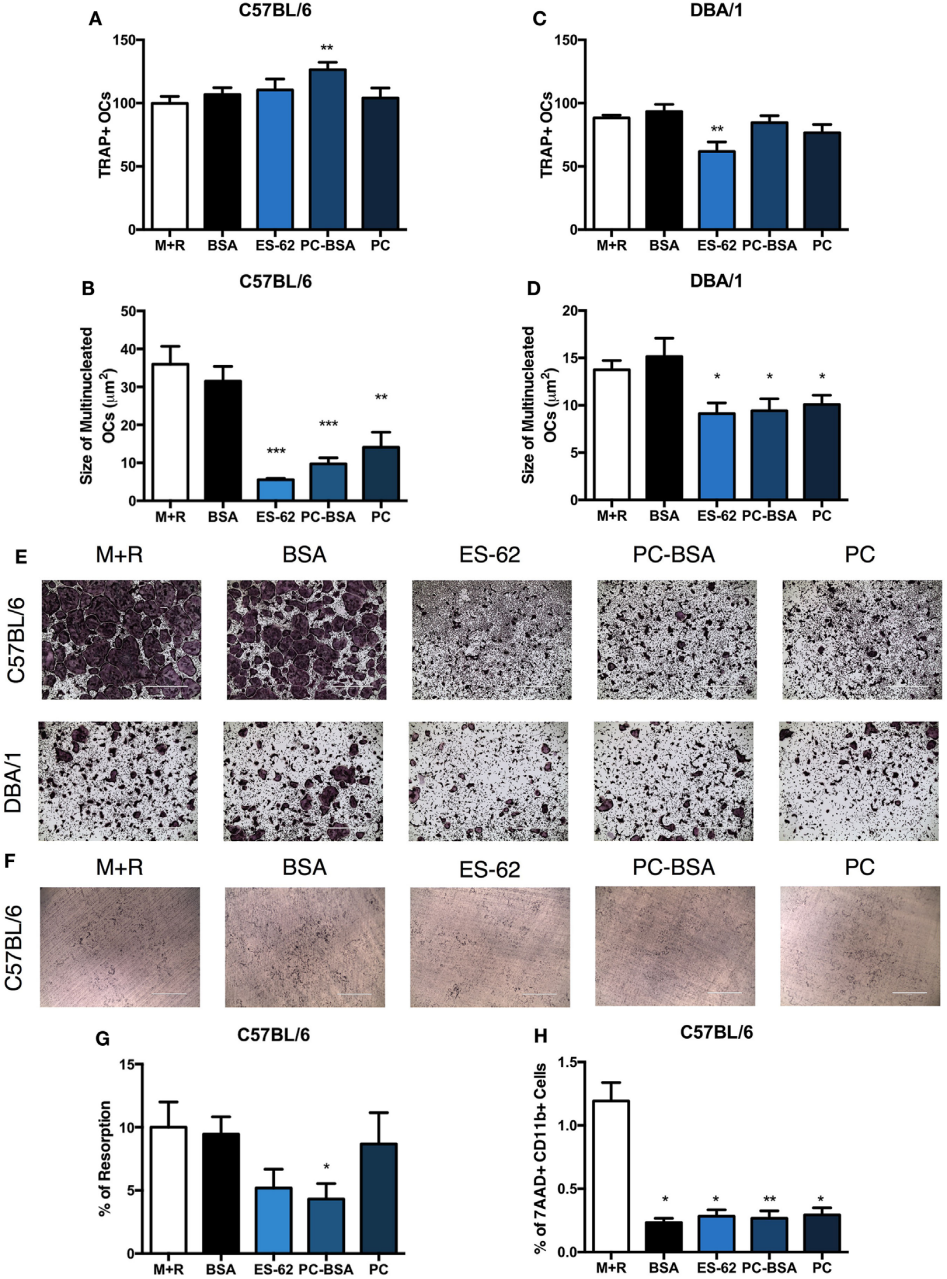
ES-62, and PC inhibit the fusion of OCs *in vitro*. Non-adherent bone marrow (BM), from healthy naïve C57BL/6 and DBA/1 mice was harvested and cultured for 4 days with 30 ng/ml M-CSF and 50 ng/ml RANKL, at which point, medium was removed and cells were incubated in fresh medium or medium containing 50 µg/ml of ES-62, PC, PC conjugated to BSA (PC-BSA), or BSA. OC differentiation at day 5 was assessed by TRAP staining and ImageJ was employed to measure the number and size of multinucleated OCs with >3 nuclei present **(A–D)**. Representative images of experimental conditions are provided [**(E)** scale bar = 1,000 µm]. To assess the ability of ES-62 to inhibit OC bone resorption, naïve C57BL/6 non-adherent BM was cultured on dentine slices with BSA, ES-62, PC-BSA, or PC treatment commencing from day 4 until day 14 when resorption was assessed and representative images are provided [**(F,G)** scale bar = 400 µm]. The effect following 24 h pre-treatment of ES-62, BSA, PC-BSA, or PC on cell viability was assessed using 7AAD^+^CD11b^+^ cells by flow cytometry **(H)**. Data are pooled from four [**(A–D)**; ES-62 treatment was only performed in two of these experiments]; two [**(G)**: ES-62 treatment reduced the capacity of the cells to resorb bone in both experiments but this inhibition was only significant compared to the control cells in one experiment]; or three **(H)** independent experiments performed in triplicate and presented as mean ± SEM. Data were analyzed using one-way ANOVA with Fisher’s LSD post-test to compare experimental conditions to M + R, where *p < 0.05, **p < 0.01, and ***p < 0.001.

To directly address the mechanisms involved in ES-62-mediated modulation of OC responses, BM was cultured with M-CSF and RANKL for 5 days and naïve BM from both DBA/1 and C57BL/6 mouse strains was employed, the latter to allow us to examine the potential for differences in OC differentiation and functional maturation between arthritis-prone and more resistant strains, respectively (28). Addition of ES-62 at day 4 did not inhibit OC differentiation in C57BL/6 animals, but significantly inhibited OC fusion that generates large, multinucleated cells in a dose-dependent manner, with maximum inhibition at the highest concentration tested, 50 µg/ml (data not shown and Figures 3A,B,E). While addition of ES-62 to DBA/1 cultures likewise significantly decreased the size of the resulting TRAP^+^ OCs, it also reduced the numbers of OCs differentiated from the BM of naive DBA/1 mice (Figures 3C–E).

PC (PC chloride) and PC conjugated to BSA (PC-BSA) were also tested in OC differentiation assays to determine whether the immunomodulatory PC-component of ES-62 was responsible for this novel effect on OCs. In the arthritis-resistant C57BL/6 animals, PC-BSA, but not PC, significantly increased the number of OCs in culture, yet PC-BSA and PC treatment both generated OCs that were smaller in size than those in the M-CSF and RANKL controls (Figures 3A,B). In DBA/1 animals, neither PC-BSA nor PC had an effect on the number of OCs generated but like ES-62, both significantly reduced the fusion of OCs resulting in smaller TRAP^+^ OCs (Figures 3C–E). BSA was used as a control for PC-BSA treatment and was shown to have no effect on the differentiation and functional maturation of OCs in either C57BL/6 or DBA/1 cultures (Figures 3A–E). Representative images are provided to demonstrate the inhibition in OC fusion observed in both C57BL/6 and DBA/1 BM cultures (Figure 3E).

The bone resorption assay was then utilized to understand how ES-62 and the PC-containing molecules were affecting the functional capacity of the OCs undergoing reduced cell fusion during *in vitro* osteoclastogenesis. This showed that ES-62 (albeit not significantly relative to the M + R control) and PC-BSA reduced the levels of bone resorption induced compared to those in M-CSF plus RANKL controls (±BSA, Figures 3F,G). These effects on osteoclastogenesis were not due to reduced cell viability as 7AAD staining showed that treatment with ES-62, PC-BSA, PC, or BSA all further reduced the very low levels of dead (% of 7AAD^+^CD11b^+^) cells resulting in culture when compared to those with M-CSF plus RANKL alone (M + R; Figure 3H). Collectively, these data indicate that the immunomodulatory PC component of ES-62 is likely to be responsible for its ability to inhibit fusion and functional maturation of OCs. Moreover, the data suggest that exposure to ES-62/PC *in vitro* prevents the functional maturation of OCs derived from BM OCPs from both healthy C57BL/6 and DBA/1 mice, predominantly by recapitulating the reduced OC fusion found during *ex vivo* osteoclastogenesis of BM from CIA mice exposed to ES-62 *in vivo* (Figure 2).

### ES-62’s SMAs 11a and 12b Inhibit Osteoclast Differentiation and Functional Maturation

**Figure 4:**
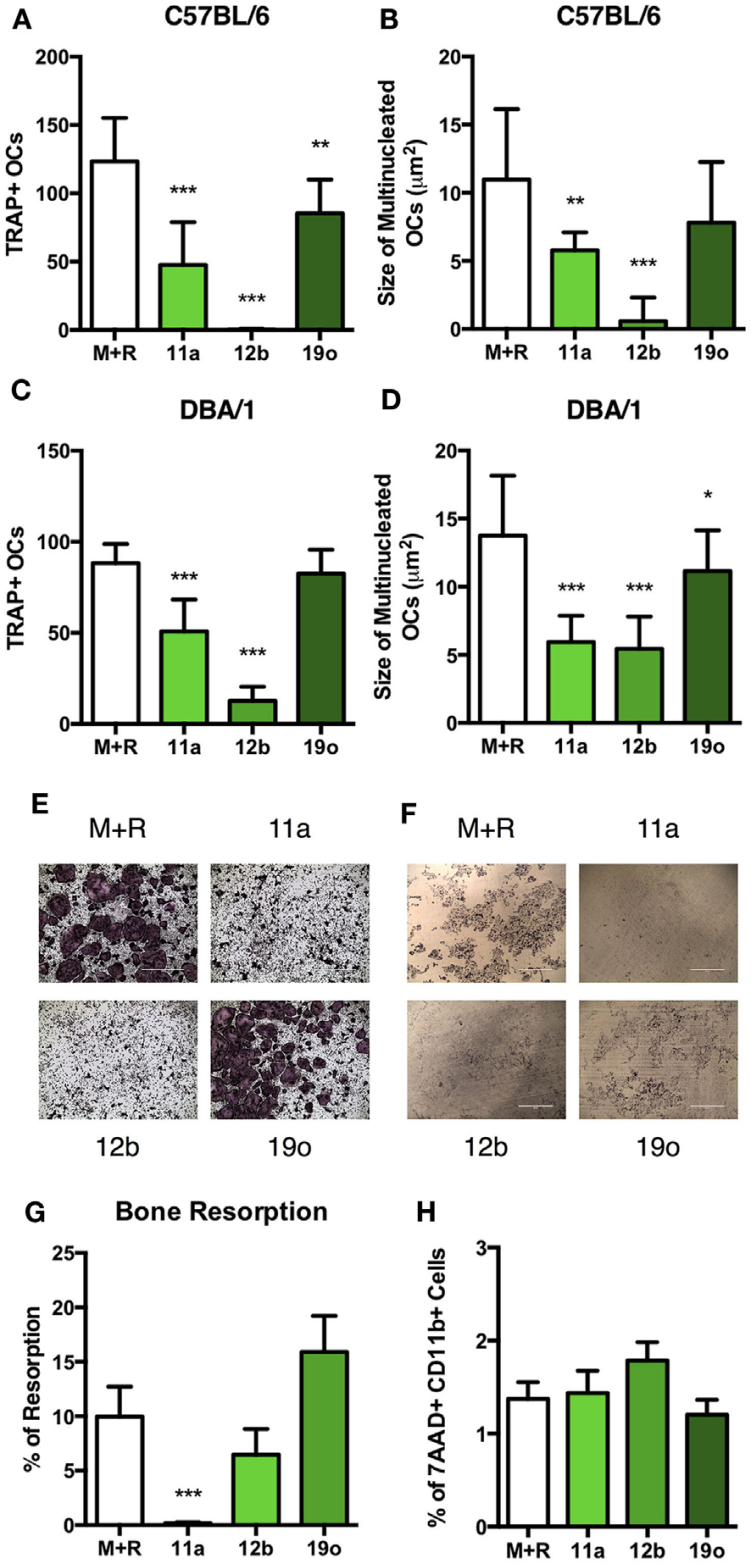
SMAs of ES-62 inhibit the differentiation and function of OCs. Non-adherent bone marrow (BM) from naïve C57BL/6 and DBA/1 mice was harvested and cultured for 4 days with 30 ng/ml M-CSF and 50 ng/ml RANKL, at which point medium was refreshed and cells were incubated in medium or medium containing 5 µg/ml SMA 11a, 12b, or 19o. OC differentiation was assessed at day 5 by TRAP staining and Image J was employed to measure the number and size of OCs present **(A–D)**. Representative images of TRAP-stained SMA-treated OCs are provided [**(E)** scale bar = 1,000 µm]. The functional capacity of SMA-treated C57BL/6 OCs was assessed on dentine slices after 14 days, where SMAs were added when medium was refreshed from day 4 onward and representative images of resorption pits provided [**(F,G)** scale bar = 400 µm]. OC precursor cell death following 24-h treatment with SMAs using 7AAD staining was measured **(H)**. Data are from three experiments performed in triplicate and presented as mean ± SD. One-way ANOVA with Fisher’s LSD post-test was used to analyze experimental conditions compared to M + R, where *p < 0.05, **p < 0.01, and ***p < 0.001. In **(G)**, 19o is not significantly different to M + R but is significantly different to 11a (*p* < 0.001) and 12b (*p* < 0.05).

The PC moiety of ES-62 has been used as the structural starting point to generate a library of drug-like synthetic SMAs that mimic the immunomodulatory capabilities of ES-62: two of these, SMAs 11a and 12b recapitulate the anti-inflammatory and bone protective effects of ES-62 and are successful in preventing development and progression of joint damage in CIA (18–20). Thus, we investigated the effect of these compounds on *in vitro* OC differentiation, with addition of SMAs 11a or 12b (at 5 µg/ml) on day 4. SMA 19o was used as a negative control for SMAs 11a and 12b, as in previous studies this SMA failed to mimic the protective immunomodulatory capabilities of ES-62, either *in vivo* or *in vitro* (18, 29). In C57BL/6 and DBA/1 cultures, SMAs 11a and 12b significantly reduced both the number and the size of differentiated OCs, compared to M + R exposure alone while, by comparison, SMA 19o exhibited little or no effect (Figures 4A–D). Representative images of SMA-treated C57BL/6 OCs are provided to highlight the reduction in the size of OCs compared to those produced following culture with M + R (Figure 4E). Furthermore, assessment of the ability of the SMAs to modulate OC function in the bone resorption assay showed that while SMA 19o, if anything, tended to promote bone resorption, SMA 11a and, to a lesser extent, 12b (albeit only significantly with respect to the SMA 19o control) inhibited bone resorption (Figures 4F,G).

Due to their therapeutic potential, we wished to ensure that the effects of the SMAs did not reflect induction of cell death: importantly analysis of cell viability showed that the % of 7AAD^+^CD11b^+^ cells in SMA-treated cultures is not increased relative to M + R treatment alone (Figure 4H). Therefore, the effects of SMAs on OC differentiation and functional maturation are not due to cell death, findings consistent not only with our previous reports on SMA-treated macrophages (18, 19), but also with the effects of ES-62, PC-BSA, and PC observed in Figure 3H.

### SMAs 11a and 12b Alter mRNA Transcription in OCs and Induce an Anti-Oxidant Response

**Figure 5:**
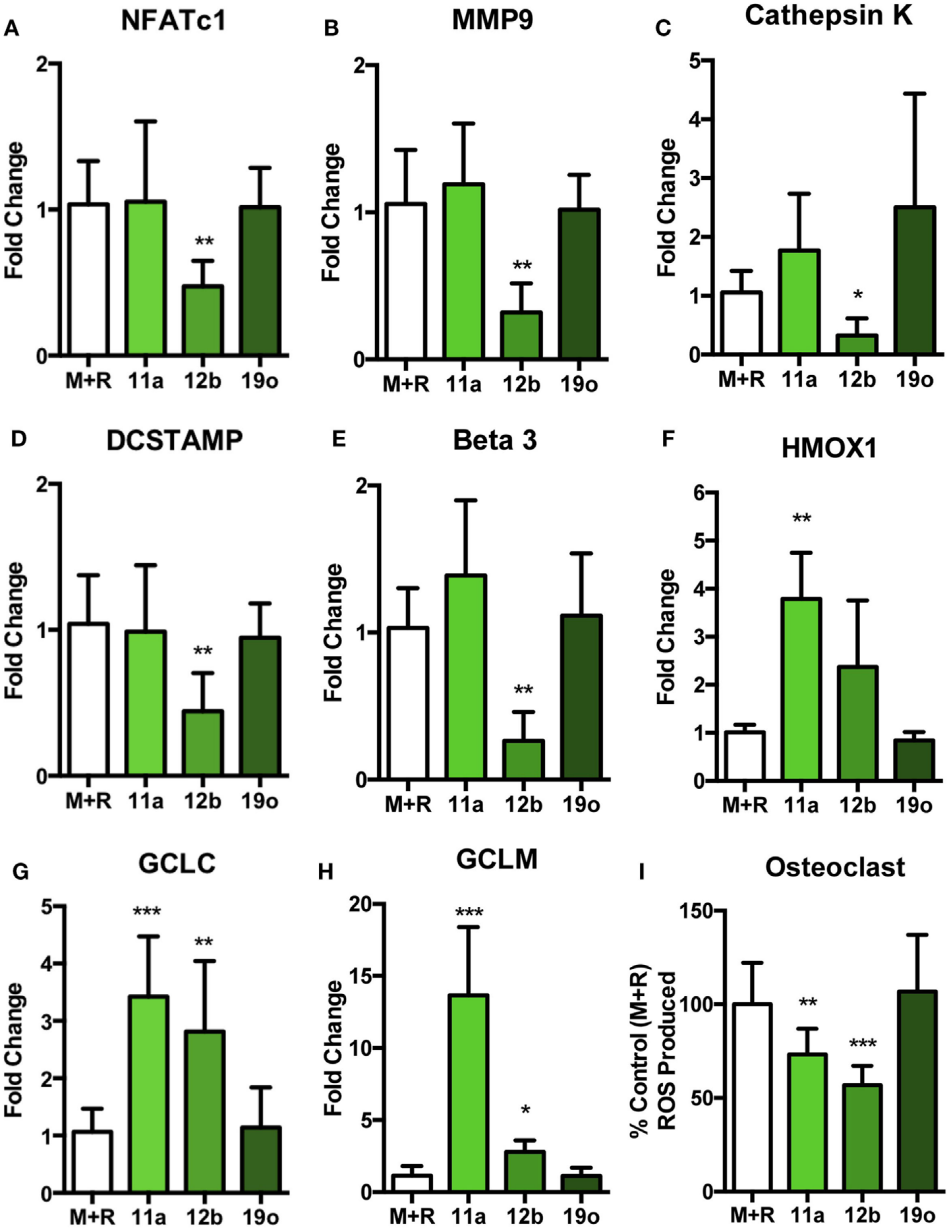
SMA 12b downregulates OC-specific gene transcription and induces an antioxidant response during OC differentiation *in vitro*. Non-adherent BM, from C57BL/6 mice, was harvested and cultured for 4 days with 30 ng/ml M-CSF and 50 ng/ml RANKL (M + R), at which point medium was refreshed and cells were incubated in medium or medium containing 5 µg/ml of SMAs 11a, 12b, or 19o. At day 5 of culture, OCs were lysed, mRNA extracted, and cDNA synthesized. Expression of *nfatc1* **(A)**, *mmp9* **(B)**, *ctsk* **(C)**, *dc-stamp* **(D)**, *itgb3* **(E)**, *hmox-1* **(F)**, *gclc* **(G)**, *and gclm* **(H)** mRNA was determined by qRT-PCR to assess the phenotypic profile of OCs. Likewise, on day 5 of culture, following the 24-h treatment with SMAs, OCs **(I)** were stained using the fluorescent stain DCF-DA to measure the presence of reactive oxygen species (ROS). Data are collated from three experiments performed in triplicate, normalized to the M + R control and presented as mean ± SD. One-way ANOVA with Dunn’s post-test was used to analyze experimental conditions compared to M + R, where *p < 0.05, **p < 0.01, and ***p < 0.001.

To identify the mechanisms underlying the observed SMA-mediated effects, the inhibition of OC differentiation by SMA 11a or 12b was further explored by examination of expression of key regulators of OC differentiation and function on cells that had been in culture for 5 days (Figures 5A–E). Interestingly, despite inhibiting differentiation and resorption, SMA 11a failed to have any effect on mRNA levels of the OC-specific genes investigated (Figures 5A–E), suggesting that this SMA utilizes another mechanism to inhibit functional differentiation of OCs. By contrast, SMA 12b was able to decrease the mRNA levels of a number of essential OC genes (Figures 5A–E). For example, the master regulator of osteoclastogenesis, NFATc1, was decreased following SMA 12b exposure (Figure 5A) and indeed, inhibition of this transcription factor by SMA 12b is likely to account for its effects on expression of the key markers of OC differentiation, MMP9, cathepsin K, DC-STAMP, and ß3 (Figures 5B–E) (10, 30) and perhaps explains its dramatic effects on the differentiation and fusion of TRAP^+^ OCs. As expected, SMA 19o generally had little or no effect in this analysis.

Previously, SMA 12b was shown to require NRF2 for its inhibition of pro-inflammatory cytokine release by macrophages (19), particularly that of IL-1β which also plays a key role in promoting osteoclastogenesis and bone resorption (31). As SMA 12b-mediated protection appeared to reflect induction of NRF2-regulated anti-oxidant genes (19) and NRF2 is a known inhibitor of OC differentiation (32), we next investigated the effect of the SMAs on the induction of NRF2 response genes in day 5 OCs. This showed that both SMA 11a and 12b, but not 19o, increased mRNA for heme oxygenase-1 (HMOX1), GCLC, and GCLM (Figures 5F–H). This dramatic increase in anti-oxidant response gene mRNA was mirrored by a significant reduction in the level of ROS produced by OCs following exposure to SMA 11a or 12b but not 19o (Figure 5I). As M-CSF and RANKL, individually and in combination, stimulate a ROS response that is required for the differentiation of mature OCs (11, 12), this inverse increase in anti-oxidant response genes and decrease in OC ROS production following SMA treatment provides a potential mechanism for the ability of both SMA 11a and 12b to inhibit OC fusion and function despite the failure of SMA 11a to alter the levels of markers associated with driving osteoclastogenesis. The combination of decreased ROS and regulators of OC differentiation presumably explains the increased efficacy of SMA 12b (relative to 11a) in suppressing functional maturation of OCs, although 11a was more effective at preventing bone resorption.

## Discussion

It has been widely established that helminths are capable of skewing immune responses in order to improve their survival and prevent expulsion from the host and that this can impact on other infections as well as medical conditions associated with aberrant inflammatory responses (33). In the context of their potential protection against arthritis, helminths promote a strong Th2 immune response that is thought to counter the Th1/17 responses that dominate this disease (34). However, previously, our work has demonstrated that the helminth product ES-62 disrupts the IL-17 network in arthritis models without promoting a Th2 phenotype: rather, by resetting homeostatic regulatory mechanisms (regulatory B cell and IL-22-dependent synovial fibroblast tissue repair responses), ES-62 acts to interfere with a number of the key pathological processes required to drive inflammation and disease (14, 17, 22, 35). Thus, this single ES product adopts a mechanism of action distinct from that commonly observed with live helminth infections, but that effectively redirects the immune system away from its state of hyper-responsiveness.

Herein, we now show that ES-62 also protects against the development of arthritis in the CIA model by modulating the progenitor populations that directly impact on bone remodeling in arthritic disease. Thus, consistent with its ability to reset homeostatic regulation, ES-62 exhibited an ability to return the levels of HSC and OC BM progenitors found in arthritic animals toward those found in healthy, “Naïve” animals and also to skew the RANK/OPG/RANKL axis such that OCPs would be less responsive to pathogenic signaling and result in less cathepsin K^+^ OCs differentiating in the inflamed joint. Furthermore, in line with the finding that OCPs in the BM are increased in the SKG spontaneous model of arthritis (24), we witnessed an increase in the monocyte and OCP populations in animals with CIA whilst treatment of these animals with ES-62 returned the numbers of monocytes, and potentially OCPs back to the levels found in healthy animals. At the same time, the expression of CD115—necessary for OC differentiation—on both of these progenitor populations was decreased following ES-62 treatment. Perhaps even more impressively, ES-62-treated CIA animals had an altered profile of functional OC maturation *ex vivo* compared to PBS-treated controls, suggesting that even limited exposure to ES-62 *in vivo* could have a lasting impact on the potential for pathogenic osteoclastogenesis in arthritis. With regards to how ES-62 modulates the progenitor populations in the BM, it is known that this worm product subverts TLR4 and MyD88 signaling to enact its immunomodulatory effects on cells (36). Interestingly, expression of TLR4, and MyD88 is required for HSC differentiation to myeloid cells following LPS stimulation (37). Thus, MyD88 may serve as a potential target for ES-62 in resetting baseline levels of HSCs, and OCPs, with this molecule providing the first example of a defined helminth ES product rewiring bone-remodeling progenitor populations *in vivo* to protect against the development of musculoskeletal disease.

In addition to the effects of ES-62 *in vivo*, the worm product directly inhibits OC differentiation *in vitro*: this ability is reminiscent of previous studies showing that a distinct glycoprotein within crude ES from the human tapeworm *Spirometra erinaceieuropaei* was able to inhibit *in vitro* OC differentiation and reduce inflammatory cytokine production although the molecular mechanisms underlying these effects were not investigated (38). More recently, Chen et al. (34) and Sarter et al. (39) both demonstrated instances where live worm infections were capable of reducing inflammatory disease in murine arthritis models, thereby resulting in fewer OCs at the inflamed joint (34, 39), with the latter group going on to demonstrate that *Heligmosomoides polygyrus* (H)ES was capable of inhibiting the differentiation of OCs *in vitro*, although again no mechanistic information was provided (39). While, similar concentrations of ES-62 and HES (50 µg/ml) have been used to inhibit OC differentiation, *in vitro* (39), HES contains the full complement of ES products from *H. polygyrus* [likely hundreds in number (40)] and it is possible that only one or two components may exert similar effects to ES-62. This suggests that the active ingredient(s) in HES may be more potent than ES-62. However, in contrast to HES-induced OC inhibition, where the parasite products were present throughout the culture, ES-62 and the SMAs exhibited an inhibitory effect on cells that had already been exposed to M-CSF and RANKL for 4 days. Thus, ES-62 and the SMAs were able to prevent the fusion and maturation of OCs even at a late stage of differentiation.

The differential effects of ES-62 and the SMAs *in vitro* with respect to differentiation of OCs from BM derived from naïve C57BL/6 mice may simply reflect the higher (~10-fold) molar concentrations of the SMAs relative to that of the active PC moiety of the parent ES-62 structure. Moreover, the SMAs contain a sulfonyl functional group in place of the phosphate group of the PC-containing molecules used in this study, which by providing additional stability may explain the relative potency of the SMAs (especially with respect to PC alone) in inhibiting OC differentiation and functional maturation. Nevertheless, as we have found in our previous CIA studies (16, 17), ES-62 appears to exhibit more potent effects under inflammatory conditions *in vivo* relative to its direct actions on “normal” isolated cells *in vitro* and this seems to reflect that, rather than suppressing responses *per se*, ES-62 acts to downregulate aberrant pathological signals to homeostatically restore steady-state responses. Alternatively, *in vivo*, ES-62 may target a cell network or induce systemic cellular or mediator effects that we have not yet recapitulated *in vitro*. Moreover, although we have generally found ES-62 to maximally modulate myeloid cell responses at 1–2 µg/ml *in vitro* [e.g., Ref. (18, 19)], we have previously shown the worm product to exhibit differential effects on naïve and activated B cell responses *in vitro* when stimulated with low or high concentrations (41). Overall, this suggests that ES-62 affects particular responses in a concentration and cell-dependent manner.

Our previous studies revealed that there are some differences in mechanism of action between the structurally similar SMA 11a and 12b despite both being modeled on the PC component of ES-62. Thus in CIA, SMA 11a preferentially targets IL-17 signaling (17), while SMA 12b predominantly targets NRF2 to inhibit, the NLRP3 inflammasome, and IL-1β (19). Furthermore, microarray analysis on BM-derived macrophages stimulated with SMA 12b highlighted several pathways that were affected following SMA treatment and, of importance to this study, identified a number of osteoclastogenic targets. These included NFATc1 and c-Fos, which are both required for the differentiation of OCs (10, 19, 42) and reflecting this, in this study, SMA 12b downregulated mRNA levels of the main regulator of OC differentiation, NFATc1. The downregulation of NFATc1 mRNA levels likely resulted in the observed effects on the expression of mRNA for genes required for OC differentiation and function, such as DC-STAMP, cathepsin K, and MMP9. Perhaps surprisingly, SMA 11a, unlike SMA 12b, was unable to modulate OC-related mRNA transcription in this study. Our previous data on macrophages also indicated that exposure to SMA 12b but not 11a for 4 h promoted NRF2 signaling (19) but our further studies now suggest that 11a also induces anti-oxidant responses, albeit this is not seen until ~24 h (unpublished data). Consistent with this, both SMAs were able to generate an anti-oxidant response in the present work as shown by increased HMOX-1, GCLC, and GCLM gene expression by 24 h. NRF2 is a negative regulator of osteoclastogenesis as *nrf2^−/−^* BM produces higher numbers of OCs compared to WT BM as a result of increased ROS production (43), which is utilized by OCs to enhance differentiation following both M-CSF and RANKL stimulation (11, 12). Both SMA 11a and 12b increase expression of these NRF-2-regulated anti-oxidant genes that inhibit ROS production resulting from M-CSF plus RANKL stimulation, suggesting that their ability to generate a potent and robust anti-oxidant response prevents the differentiation of mature OCs. Although, we have not definitively shown that the SMAs utilize NRF2 to drive an anti-oxidant response to inhibit OC differentiation, future studies exploiting *nrf2^−/−^* mouse models might provide valuable insight into the role the anti-oxidant response plays in SMA-mediated inhibition of OC differentiation and functional maturation (32, 43).

Interestingly, inhibition of osteoclastogenesis by ES-62 and the SMAs was achieved in BM from naïve animals from both arthritis-prone and more resistant strains of mice. CIA is reproducibly induced in DBA/1 mice with approximately 80–100% incidence while CIA incidence in C57BL/6 mice can range between 0 and 60% (28). Thus, in spite of the relative differences in disease susceptibility between these strains, ES-62 and the SMAs were able to inhibit not only the fusion of smaller TRAP^+^ OCPs into large multinucleated OCs, but also the bone resorption activities of such OCs in both cases. This may suggest a therapeutic potential even in humans highly susceptible to developing disease.

In summary, we show that exposure to the parasitic helminth product ES-62 reduces the pool of progenitors in the BM of CIA animals and results in an altered course of monocyte/OC differentiation. Mechanistically, this is because ES-62, and SMAs based on the active PC moiety of ES-62, can inhibit the differentiation and fusion of OCs *in vitro* and the SMAs are capable of inducing NRF2-related anti-oxidant genes in OCs. Together these data are representative of a novel area of research linking the immunomodulatory capabilities of parasitic helminths and osteoimmunology that has perhaps previously been overlooked. Therapeutically, this could have ramifications for RA, as well as other OC-driven pathologies, as ES-62 not only prevents the inflammatory pathology of CIA, but also rewires the effector cells to a “safe” phenotype that could provide longer lasting benefits beyond current therapeutic interventions.

## Ethics Statement

All experiments employing animals were approved by, and conducted in accordance with, the Animal Welfare and Ethical Review Board of the Universities of Strathclyde and Glasgow and UK Home Office Regulations and Licenses PIL I518666F7, PPL 60/4314, PPL P8C60C865, PIL 1675F0C46, and PIL ICEBDB864.

## Author Contributions

JD, FL, MP, AT, JC, and AK performed the experiments for the study that JD, MH, and WH conceived. JD and FL manufactured ES-62 and CS produced ES-62’s SMAs. JD, MH, and WH wrote the paper and all authors were involved in reviewing and revising the manuscript and have approved the final version.

## Conflict of Interest Statement

The authors declare that the research was conducted in the absence of any commercial or financial relationships that could be construed as a potential conflict of interest.
